# Development of a machine learning-based multimode diagnosis system for lung cancer

**DOI:** 10.18632/aging.103249

**Published:** 2020-05-23

**Authors:** Shuyin Duan, Huimin Cao, Hong Liu, Lijun Miao, Jing Wang, Xiaolei Zhou, Wei Wang, Pingzhao Hu, Lingbo Qu, Yongjun Wu

**Affiliations:** 1 College of Public Health, Zhengzhou University, Zhengzhou 450001, China; 2 The First Affiliated Hospital of Zhengzhou University, Zhengzhou 450001, China; 3 Henan Provincial Chest Hospital, Zhengzhou 450001, China; 4 Department of Biochemistry and Medical Genetics, University of Manitoba, Winnipeg, MB R3E 3N4, Canada; 5 Henan Joint International Research Laboratory of Green Construction of Functional Molecules and Their Bioanalytical Applications, Zhengzhou 450001, China; 6 The Key Laboratory of Nanomedicine and Health Inspection of Zhengzhou, Zhengzhou 450001, China

**Keywords:** machine learning, lung cancer, multidimensional variables, multimode diagnosis

## Abstract

As an emerging technology, artificial intelligence has been applied to identify various physical disorders. Here, we developed a three-layer diagnosis system for lung cancer, in which three machine learning approaches including decision tree C5.0, artificial neural network (ANN) and support vector machine (SVM) were involved. The area under the curve (AUC) was employed to evaluate their decision powers. In the first layer, the AUCs of C5.0, ANN and SVM were 0.676, 0.736 and 0.640, ANN was better than C5.0 and SVM. In the second layer, ANN was similar with SVM but superior to C5.0 supported by the AUCs of 0.804, 0.889 and 0.825. Much higher AUCs of 0.908, 0.910 and 0.849 were identified in the third layer, where the highest sensitivity of 94.12% was found in C5.0. These data proposed a three-layer diagnosis system for lung cancer: ANN was used as a broad-spectrum screening subsystem basing on 14 epidemiological data and clinical symptoms, which was firstly adopted to screen high-risk groups; then, combining with additional 5 tumor biomarkers, ANN was used as an auxiliary diagnosis subsystem to determine the suspected lung cancer patients; C5.0 was finally employed to confirm lung cancer patients basing on 22 CT nodule-based radiomic features.

## INTRODUCTION

Lung cancer is the most common cause of cancer-related death worldwide due to insidious incidence, high metastasis, and poor prognosis [1]. As reported by the Annual Report of America in 2018, the five-year survival rate of lung and bronchus cancer ranged from 55.1% (stage I) to 4.2% (stage IV) for cases that were diagnosed from 2007 through 2013 [2]. However, only 25.3% of lung and bronchus cancer patients were diagnosed at stage I or stage II, while 66.9% of cases were diagnosed at stage III or stage IV due to the lack of an efficient early diagnostic tool for lung cancer [2]. Five-year survival analysis by stage and the examination of stage distribution indicates the potential benefits associated with early detection and treatment [2]. Thus, it is essential to develop a novel early diagnostic strategy, which contributes to enhancing clinical therapeutic efficacies for lung cancer.

Nowadays, chemical diagnosis, imaging diagnosis, cell and histocytological diagnosis are the primary diagnostic methods of lung cancer [3]. Among them, computed tomography (CT)-based imaging diagnosis is the primary tool to detect lung cancer at early stages [4–6]. The results of the National Lung Screening Trial confirmed that low-dose CT (LDCT) adopted in the high-risk group could reduce the mortality rate of lung cancer by 20% compared with chest X-ray [6]. Several other studies also demonstrated that CT scans should be implemented for the high-risk groups, but not for the general population, to detect early lung cancer, which could decrease the radiation hazard and financial costs [7–9]. However, it is a difficult task to identify the high-risk group for lung cancer. At present, the definition of the high-risk group for lung cancer is controversial, which is mainly assessed by age and smoking status [7]. Evidence showed that lung cancer could also be indicated by other epidemiological characteristics and clinical symptoms such as the family history of cancer and hemoptysis [7, 9, 10].

Indeed, CT provides effective early diagnostic information of lung cancer from a macroscopic perspective, which can clearly locate the nodule sites and indicate the metastasis. It is known that radiologists distinguish the benign from malignant nodules by their size, shape, density, and other characteristics [11]. However, CT images are difficult to be analyzed manually, which requires radiologists to have excellent reading skills, especially for the diagnosis of small and isolated pulmonary nodule [12, 13]. It is reported that the false positive rate of LDCT screening for lung cancer is as high as 96.4% [6]. Therefore, the diagnostic efficiency of CT for lung cancer needs to be further improved. On one hand, it is necessary to develop a method that can effectively distinguish benign from malignant CT nodules. At present, many scholars try to extract radiomic features of CT nodules and establish models to achieve the intelligent identification of benign and malignant nodules [12, 14, 15]. On the other hand, there is an urgent need to seek an auxiliary means, which can enhance the diagnostic efficiency of lung cancer in combination with CT. As we know, tumor markers have been widely used in the detection of lung cancer in recent years, such as progastrin-releasing peptide (ProGRP), vascular endothelial growth factor (VEGF), carcinoembryonic antigen (CEA), cytokeratin 19 fragment (CYFRA21-1) and neuronspecific enolase (NSE) [16, 17]. Previous studies confirmed that the risk model constructed with these tumor markers could enhance the early diagnosis of lung cancer [18, 19]. Certainly, tumor markers in serum provide microscopic molecular information related to the occurrence and progression of cancer, which points out a new direction for the early detection of lung cancer [16, 20]. In addition, blood sampling, minimally invasive and repeatable, can be easily performed, making serum an excellent matrix for lung cancer diagnosis [20, 21]. Thus, the combination of tumor markers and the features of CT nodules, which offers microscopic molecular information and macroscopic imaging information, is supposed to be an ideal strategy for lung cancer diagnosis at early stages [22]. However, medical data in current studies are complex, which cannot be processed adequately by traditional statistical methods. Especially, parameter analysis and information mining are challenging tasks [23]. Machine learning based on data mining technology can extract valuable knowledge and information from a large number of incomplete and noisy data, which may be suited for this work [24]. Recent studies have demonstrated that the application of machine learning significantly improves metastases detection in lymph nodes, Ki67 scoring in breast cancer, Gleason grading in prostate cancer, and tumor-infiltrating lymphocyte scoring in melanoma [25]. Furthermore, deep machine learning models are able to predict the changes of some tumor markers in lung, prostate, gastric, and colorectal cancer [25]. Moreover, prognostic deep neural network models have been adopted in the diagnosis of lung cancer, melanoma, and glioma, which is developed based on digitized HE slides [25]. Among the various machine learning approaches, decision tree (DT) C5.0, artificial neural network (ANN), and support vector machine (SVM) have been widely applied in the development of cancer prediction models, which has resulted in making effective and accurate diagnosis [26].

In this study, C5.0, ANN, and SVM were applied to develop an efficient multilayer diagnosis system for lung cancer based on multidimensional variables. The diagnosis system integrated epidemiological characteristics, clinical symptoms, and molecular markers with CT nodule-based radiomic features, which combined micro biomarkers with macro imaging, behavior characteristics, and laboratory research with clinical diagnosis technology.

## RESULTS

### Statistical analysis of epidemiological characteristics and clinical symptoms from 842 cases in the first-layer subsystem

The comparisons of the 14 features describing the epidemiological characteristics and clinical symptoms (between the 372 lung cancer and the 470 lung benign diseases) were shown in Table 1. Statistical analysis showed that there were significant differences between the two groups (*P*<0.05) for the characteristics of age by groups, age, gender, smoking status, drinking status, history of lung infection, expectoration, bloody sputum, fever or sweating, cough and hemoptysis. And, there were no significant differences between lung cancer and lung benign groups (*P*>0.05) for chest tightness or chest pain, family history of tumor and lung cancer.

**Table 1:** Demographic characteristics of lung cancer and lung benign disease patients in the first-layer subsystem.

**Variables**	**Lung benign (n=470)**	**Lung cancer (n=372)**	**χ^2^/Z**	***P***
Age By Groups				
≤45	134	26	62.487	<0.001^*^
>45	336	346		
Age (year)	57(44-67)	60(52-67)	-3.882	<0.001^*^
Gender				
Female	213	123	13.004	<0.001^*^
Male	257	249		
Smoking Status				
No	359	210	37.649	<0.001^*^
Yes	111	162		
Drinking Status				
No	405	290	9.720	0.002^*^
Yes	65	82		
History of Lung Infection				
No	167	108	3.989	0.046^*^
Yes	303	264		
Chest Tightness or Chest Pain				
No	230	176	0.219	0.639
Yes	240	196		
Expectoration				
No	209	132	6.955	0.008^*^
Yes	261	240		
Bloody Sputum				
No	428	290	28.406	<0.001^*^
Yes	42	82		
Cough				
No	144	88	8.180	0.004^*^
Yes	326	284		
Hemoptysis				
No	432	319	5.072	0.024^*^
Yes	38	53		
Fever or Sweating				
No	280	289	31.095	<0.001^*^
Yes	190	83		
Family History of Tumor				
No	446	342	3.027	0.082
Yes	24	30		
Family History of Lung Cancer				
No	445	346	1.018	0.313
Yes	25	26		

*: Statistically significant at *P*=0.05 level.

### Demographic characteristics and serum levels of ProGRP, VEGF, CEA, CYFRA21-1, and NSE for the study subjects in the second-layer subsystem

Demographic characteristics of lung cancer and lung benign disease patients in the second-layer subsystem were presented in Table 2. There were significant differences between the two groups (*P*<0.05) for the characteristics of age by groups, smoking status, history of lung infection, expectoration, bloody sputum, fever or sweating, hemoptysis and family history of lung cancer. In contrast, there were no significant differences between lung cancer and lung benign patients (*P*>0.05) for age, gender, drinking status, chest tightness or chest pain, cough and family history of tumor. As shown in Table 3, the levels of ProGRP, VEGF, CEA, and CYFRA21-1 in the lung cancer group were higher than those in the lung benign disease group (*P*<0.05). However, there was no statistical difference in the level of NSE between the two groups (*P*>0.05).

**Table 2:** Demographic characteristics of subjects in the second-layer subsystem.

**Variables**	**Lung benign (n=157)**	**Lung cancer (n=129)**	**χ^2^/Z**	***P***
Age By Groups				
≤45	41	8	19.778	<0.001^*^
>45	116	121		
Age (year)	58(45-67)	59(52.5-66)	-1.834	0.067
Gender				
Female	65	51	0.102	0.749
Male	92	78		
Smoking Status				
No	114	70	10.390	0.001^*^
Yes	43	59		
Drinking Status				
No	133	98	3.486	0.062
Yes	24	31		
History of Lung Infection				
No	103	68	4.895	0.027^*^
Yes	54	61		
Chest Tightness or Chest Pain				
No	71	63	0.371	0.542
Yes	86	66		
Expectoration				
No	78	43	7.754	0.005^*^
Yes	79	86		
Bloody Sputum				
No	140	93	13.682	<0.001^*^
Yes	17	36		
Cough				
No	51	29	3.517	0.061
Yes	106	100		
Hemoptysis				
No	145	105	7.733	0.005^*^
Yes	12	24		
Fever or Sweating				
No	84	95	12.267	<0.001^*^
Yes	73	34		
Family History of Tumor				
No	141	110	1.358	0.244
Yes	16	19		
Family History of Lung Cancer				
No	152	117	4.740	0.029*
Yes	5	12		

*: Statistically significant at *P*=0.05 level.

**Table 3:** Comparison of the 5 tumor markers between lung cancer and lung benign diseases.

**Tumor markers**	**Lung benign (n=157) M(P25-P75)**	**Lung cancer (n=129) M(P25-P75)**	**Z**	***P***
ProGRP (pg/mL)	18.59(11.61-30.39)	27.50(15.76-44.40)	-4.298	<0.001^*^
VEGF (ng/mL)	2.25(1.38-3.42)	3.00(1.95-4.06)	-4.318	<0.001^*^
CEA(ng/mL)	2.27(1.39-4.39)	2.95(1.87-5.55)	-2.705	0.007^*^
CYFRA21-1(ng/mL)	1.50(0.77-2.15)	1.57(0.96-1.80)	-2.009	0.044^*^
NSE(ng/mL)	9.30(5.83-15.19)	8.88(5.36-15.04)	-0.727	0.467

*: Statistically significant at *P*=0.05 level.

### Statistical analysis of the 22 radiomic features extracted from lung CT nodules in the third-layer subsystem

The demographic characteristics of the subjects in the third-layer subsystem were shown in Supplementary Table 1. 22 lung CT nodule-based radiomic features were extracted from 123 lung CT nodules, which contained 64 lung benign nodules and 59 lung cancer nodules. However, the extracted lobulation grade f13 and spiculation grade f14 were 0 in both groups, which couldn't be further statistically analyzed. As shown in Table 4, statistical analysis indicated that there were significant differences between the two groups (*P*<0.05) for the radiomic features of gray mean f1, gray variance f2, gray histogram entropy f3, seven order invariant distance f4, calcification area f11, calcification area/nodule area f12, cavity number f15, contrast f18, correlation f19, energy f20, homogeneity f21 and entropy f22. However, there were no significant differences between lung CT benign and malignant nodules (*P*>0.05) for the seven order invariant distance f5, f6, f7, f8, f9, f10, cavity area f16 and cavity area/nodules area f17.

**Table 4:** Comparison of radiomic features extracted from lung CT benign and malignant nodules.

**Features**	**Lung benign (n=64) M(P25-P75)**	**Lung cancer (n=59) M(P25-P75)**	**Z**	***P***
f1	0.043(0.023-0.648)	0.198(0.137-0.347)	-8.839	<0.001^*^
f2	0.025(0.014-0.045)	0.121(0.092-0.154)	-8.890	<0.001^*^
f3	0.591(0.352-0.830)	1.722(1.237-2.367)	-8.490	<0.001^*^
f4	9.0E-4(1.0E-3-1.1E-3)	8.0E-4(7.0E-4-8.0E-4)	-7.163	<0.001^*^
f5	3.1E-8(1.3E-8-9.4E-8)	1.9E-8(7.8E-9-6.4E-8)	-1.311	0.190
f6	2.9E-12(1.5E-12-5.4E-12)	2.8E-12(1.1E-12-7.4E-12)	-0.420	0.674
f7	2.7E-12(7.8E-13-5.6E-12)	1.6E-12(2.6E-13-4.0E-12)	-1.741	0.082
f8	1.5E-26(-2.8E-24-2.5E-24)	4.0E-26(-8.7E-26-3.8E-24)	-1.306	0.192
f9	-3.4E-16(-1.7E-15-5.0E-16)	-6.2E-19(-8.7E-16-6.1E-16)	-1.802	0.072
f10	9.3E-26(-4.7E-24-2.1E-24)	-9.7E-27(-1.3E-24-2.8E-24)	-0.197	0.843
f11	36.50(6.25-106.50)	814(453-1722)	-8.714	<0.001^*^
f12	0.16(0.05-0.30)	0.54(0.36-0.68)	-7.423	<0.001^*^
f15	0.00(0.00-0.00)	0.00(0.00-1.00)	-0.819	<0.001^*^
f16	0.00(0.00-6.75)	1.00(-2.00-17.00)	-0.583	0.560
f17	0(0-3.4E-2)	7.0E-5(-1.7E-4-1.4E-3)	-1.298	0.194
f18	132.63(90.59-220.19)	450.39(343.46.76-617.20)	-8.368	<0.001^*^
f19	0.956(0.945-0.963)	0.971(0.963-0.976)	-6.202	<0.001^*^
f20	0.849(0.784-0.913)	0.484(0.322-0.645)	-8.657	<0.001^*^
f21	0.944(0.919-0.966)	0.834(0.759-0.890)	-8.115	<0.001^*^
f22	3.088(2.633-3.576)	5.316(4.342-6.6930	-8.409	<0.001^*^

*: Statistically significant at *P*=0.05 level.

### Development of machine learning models

As shown in Table 5, machine learning models were constructed to distinguish lung cancer from lung benign diseases. 14 epidemiological characteristics and clinical symptoms of 638 samples, including 296 lung cancer and 342 lung benign diseases, were used as input features to develop the models of C5.0-1, ANN-1, and SVM-1 in the training set. The accuracies of C5.0-1, ANN-1, and SVM-1 models in the training set were 79.78%, 73.04%, and 77.27%, respectively. 204 samples, including 76 cases with lung cancer and 128 lung benign diseases, were used as the testing set to verify the effect of the three models. The accuracies of the C5.0-1, ANN-1, and SVM-1 models in the testing set were 69.12%, 71.57%, and 65.20%, respectively. The 14 features mentioned above and the 5 serum tumor markers levels including ProGRP, VEGF, CEA, CYFRA21-1 and NSE from 208 patients were employed as the input variables to develop the C5.0-2, ANN-2 and SVM-2 models in the training set, which included 97 lung cancer and 111 lung benign disease patients. The accuracies of C5.0-2, ANN-2, and SVM-2 models in the training set were 97.60%, 85.58%, and 98.08%, respectively. 78 samples, including 32 lung cancer and 46 lung benign diseases, were employed to test the effect of C5.0-2, ANN-2, and SVM-2 models. The accuracies of models in the testing set were 80.77%, 89.74%, and 83.33%, respectively. 22 radiomic features were extracted from 90 lung CT nodules and adopted to train the C5.0-3, ANN-3, and SVM-3 models, which included 42 lung cancer nodules and 48 lung benign nodules. The accuracies of C5.0-3, ANN-3, and SVM-3 models in the training set were 100%, 93.33%, and 100%, respectively. 33 samples, including 17 lung cancer nodules and 16 lung benign nodules, were used to test the effect of the models. The accuracies of C5.0-3, ANN-3, and SVM-3 models in the testing set were 90.91%, 90.91%, and 84.85%, respectively.

**Table 5:** Results of machine learning models to distinguish lung cancer from lung benign diseases.

**Models**		**Training set**			**Testing set**		
		**Lung benign**		**Lung bancer**	**Lung benign**		**Lung cancer**
C5.0-1	Lung Benign	280		67	94		29
	Lung Cancer	62		229	34		47
	Total	342		296	128		76
	Accuracy		79.78%			69.12%	
ANN-1	Lung Benign	238		68	84		14
	Lung Cancer	104		228	44		62
	Total	342		296	128		76
	Accuracy		73.04%			71.57%	
SVM-1	Lung Benign	270		73	88		31
	Lung Cancer	72		223	40		45
	Total	342		296	128		76
	Accuracy		77.27%			65.20%	
C5.0-2	Lung Benign	107		1	38		7
	Lung Cancer	4		96	8		25
	Total	111		97	46		32
	Accuracy		97.60%			80.77%	
ANN-2	Lung Benign	99		18	43		5
	Lung Cancer	12		79	3		27
	Total	111		97	46		32
	Accuracy		85.58%			89.74%	
SVM-2	Lung Benign	109		2	40		7
	Lung Cancer	2		95	6		25
	Total	111		97	46		32
	Accuracy		98.08%			83.33%	
C5.0-3	Lung Benign	48		0	14		1
	Lung Cancer	0		42	2		16
	Total	48		42	16		17
	Accuracy		100%			90.91%	
ANN-3	Lung Benign	46		4	15		2
	Lung Cancer	2		38	1		15
	Total	48		42	16		17
	Accuracy		93.33%			90.91%	
SVM-3	Lung Benign	48		0	14		3
	Lung Cancer	0		42	2		14
	Total	48		42	16		17
	Accuracy		100%			84.85%	

### Effect evaluation of machine learning models

As presented in Table 6, the testing effect of the model was evaluated by sensitivity, specificity, accuracy, PPV, NPV, and AUC. The sensitivities of C5.0-1, ANN-1, and SVM-1 models were 61.84%, 81.58%, and 59.21%, respectively. The specificities were 73.44%, 65.63%, and 68.75%, respectively. The AUCs were 0.676 (95% confidence interval [CI] 0.608 to 0.740), 0.736 (95%CI 0.670 to 0.795) and 0.640 (95%CI 0.570 to 0.706), respectively. The sensitivities of C5.0-2, ANN-2, and SVM-2 models were 78.13%, 84.38%, and 78.13%, respectively. The specificities were 82.61%, 93.48%, and 86.96%, respectively. The AUCs were 0.804 (95%CI 0.698 to 0.885), 0.889 (95%CI 0.798 to 0.949) and 0.825 (95%CI 0.732 to 0.902), respectively. The sensitivities of C5.0-3, ANN-3, and SVM-3 models were 94.12%, 88.24%, and 82.35%, respectively. The specificities were 87.50%, 93.75%, and 87.50%, respectively. The AUCs were 0.908 (95%CI 0.755 to 0.980), 0.910 (95%CI 0.758 to 0.981) and 0.849 (95%CI 0.682 to 0.949), respectively. To optimize the diagnostic model, the efficiency of different models was compared using the AUC in the testing set (Supplementary Table 2). Results showed that the efficiency of the ANN-1 model was higher than C5.0-1 (*Z*=1.981, *P*=0.048) and SVM-1 (*Z*=3.283, *P*=0.001). ANN-2 model was better than C5.0-2 (*Z*=2.021, *P*=0.043), and there was no difference between ANN-2 and SVM-2 by AUC comparison (*P*>0.05). But, the sensitivity of ANN-2 (84.38%) was higher than SVM-2 (78.13%). Although there were no statistical differences by AUC comparison among ANN-3, SVM-3, and C5.0-3 (*P*>0.05), C5.0-3 had the highest sensitivity of 94.12% in the three models.

**Table 6:** Effect evaluation of machine learning models in the testing set.

**Models**	**Accuracy(%)**	**Sensitivity(%)**	**Specificity(%)**	**PPV(%)**	**NPV(%)**	**AUC(95% CI)**
C5.0-1	69.12	61.84	73.44	58.02	76.42	0.676 (0.608-0.740)
ANN-1	71.57	81.58	65.63	58.49	85.71	0.736 (0.670-0.795)
SVM-1	65.20	59.21	68.75	52.94	73.95	0.640 (0.570-0.706)
C5.0-2	80.77	78.13	82.61	75.76	84.44	0.804 (0.698-0.885)
ANN-2	89.74	84.38	93.48	90.00	89.58	0.889 (0.798-0.949)
SVM-2	83.33	78.13	86.96	80.65	85.11	0.825 (0.732-0.902)
C5.0-3	90.91	94.12	87.50	88.89	93.33	0.908 (0.755-0.980)
ANN-3	90.91	88.24	93.75	93.75	88.24	0.910 (0.758-0.981)
SVM-3	84.85	82.35	87.50	87.50	82.35	0.849 (0.682-0.949)

## DISCUSSION

Although lung cancer has no specific symptoms in its early stage, there are molecular abnormalities and imaging changes during the occurrence and development of lung cancer. The characteristic information can be captured and used for the diagnosis of lung cancer. However, there are different types of data, including descriptive epidemiological and clinical symptoms, quantitative tumor markers, and CT nodule radiomic features. Traditional statistical methods are incompetent in analyzing these data. With the development of information technology, machine learning can extract valuable knowledge and information from a large number of fuzzy, incomplete, and noisy data, which may be suitable for solving such problems. In this study, powerful machine learning models DTs, ANNs, and SVMs were employed to construct the diagnostic systems of lung cancer [28]. DTs are tree-structured schemes where the nodes represent the input variables, and the leaves correspond to decision outcomes [26]. They are widely used for classification purposes and can be intuitive [3]. ANNs are developed on the basis of biological neurons of the human brain and trained to generate an output outcome as a weighted combination of the input variables [29, 30]. They aim to solve a variety of classification or pattern recognition problems [26]. The main advantage of ANN is able to approximate any nonlinear mathematical function [31]. SVMs are based on the principle of structural risk minimization and put the data into a multidimensional space to achieve classification with a hyperplane, which have distinct advantages in solving problems such as the small sample size, nonlinear, or high dimensional pattern types [3, 31]. Every approach has its advantages and disadvantages, and it is necessary to try different methods to seek a suitable model for the diagnosis of lung cancer.

Previous studies demonstrated that screening with the use of CT in high-risk groups reduced mortality from lung cancer, but not in the general population [6–9]. The risk assessment of lung cancer involved multiple factors, which contained epidemiological characteristics and clinical symptoms [9, 32]. In this study, 14 epidemiological characteristics and clinical symptoms from 842 subjects were investigated to build C5.0-1, ANN-1, and SVM-1 models. And, the results showed that the ANN-1 model had the best performance. To our knowledge, the definitions of people at risk for lung cancer vary globally, which mainly depend on age and smoking status [6–9]. Our current model determines lung cancer by integrating multiple factors including age and smoking status, which has been proved to be an effective tool for identifying lung cancer. Moreover, epidemiological characteristics and clinical symptoms can be easily obtained by a questionnaire, which is economical and physically harmless. Therefore, the ANN-1 model constructed based on these data is recommended for the broad-spectrum screening of a large sample population in the first-layer subsystem, which contributes to screening out the high-risk group of lung cancer from patients with pulmonary diseases.

In addition, another strategy - tumor markers in the blood may further help screen the persons who are best suited for CT scan and this will help to decrease the radiation hazard and financial costs [6]. In recent years, ProGRP, VEGF, CEA, CYFRA21-1, and NSE are identified as the tumor markers of lung cancer, which are commonly adopted in clinical detection [33–35]. Increasing evidence suggests that the combined assessment of serum molecular markers can effectively discriminate lung cancer [35, 36]. According to our results, the performance of ANN-2 and SVM-2 models were superior to C5.0-2 by AUC comparison, which was established with 14 features of epidemiological and clinical data, and 5 serum tumor markers of ProGRP, VEGF, CEA, CYFRA21-1and NSE from 286 samples. And the sensitivity, specificity, accuracy, PPV, and NPV of ANN-2 were higher than SVM-2. Therefore, we propose the use of the ANN-2 model for searching suspected lung cancer patients from high-risk groups, which is named as auxiliary diagnosis subsystem. Further, only the suspected lung cancer patients are recommended to perform CT scans, which will reduce the radiation hazard and alleviate the financial burdens of CT scans. However, CT scan also faces other challenges such as over-diagnosis and high false-positive rate [6, 8]. To overcome these obstacles, the benign and malignant lung nodules on CT images were analyzed [37]. 22 radiomic features were extracted from 123 lung CT nodules, based on which, ANN-3, C5.0-3, and SVM-3 models were developed. All models showed good performance in terms of sensitivity, specificity, accuracy, PPV, NPV, and AUC. In particular, the AUCs of ANN-3 and C5.0-3 were up to 0.9. Although there were no statistical differences by AUC comparison among the three models, the C5.0-3 had the highest sensitivity of 94.12%. Hence, the C5.0-3 model is recommended for distinguishing lung malignant from benign nodules, which can be utilized for the intelligent diagnosis of lung cancer.

Based on our results, we propose an efficient diagnostic strategy for lung cancer, which contains a three-layer system structure. The first layer that broad-spectrum screening subsystem is constructed based on 14 epidemiological characteristics and clinical symptoms using an ANN model for screening high-risk groups from patients with pulmonary diseases. The second layer is an auxiliary diagnosis subsystem built on epidemiological characteristics, clinical symptoms, and 5 serum tumor markers of lung cancer, including ProGRP, VEGF, CEA, CYFRA21-1, and NSE, with an ANN model for searching suspected lung cancer patients from high-risk groups. The third layer that intelligent diagnosis subsystem is developed based on 22 lung CT nodule-based radiomic features using a C5.0 model for the further confirmation of lung cancer patients. The patients with lung cancer will be diagnosed step by step, so as to reduce the radiation hazard, over-diagnosis, and financial costs. This strategy can be used for the on-site screening and clinical diagnosis of the high-risk population.

## MATERIALS AND METHODS

### Collection of clinical samples

Epidemiological characteristics and clinical symptoms of 372 lung cancer and 470 lung benign patients were collected from the First Affiliated Hospital of Zhengzhou University. All the subjects were surveyed through a questionnaire made up of 14 epidemiological characteristics and clinical symptoms, which included age, age grouping, gender, smoking history, drinking history, history of lung infection, family history of tumor and lung cancer, chest tightness or chest pain, expectoration, bloody sputum, cough, hemoptysis, fever or sweating. Smokers were defined as people who smoked one or more cigarettes per day for more than six months. The alcohol-drinkers were defined as drinking alcohol at least 12 times a year. A total of 129 patients with lung cancer and 157 patients with benign alterations of the 842 subjects donated the serum samples, among which the pulmonary CT images of 59 patients with lung cancer and 64 patients with benign alterations were simultaneously collected from the Radiology Department of the First Affiliated Hospital of Zhengzhou University. All patients with lung cancer were included according to the following inclusion criteria: (1) Patients were confirmed by the clinical diagnosis of pathology; (2) without undergoing surgical resection, chemotherapy, or radiotherapy; (3) without previous other organ tumors. Patients were excluded with significant organ function failure, pregnant, or lactating. Patients with histologically confirmed lung cancer included lung squamous cell carcinoma, lung adenocarcinoma, small cell carcinoma, and so on. Lung benign diseases included pneumonia, chronic obstructive pulmonary disease, pulmonary fibrosis, tuberculosis, and so on. The study protocol was approved by the Ethics Committee at the University of Zhengzhou. Permission for data and sample collection was obtained from the patients or their relatives.

### Measurement of 5 serum tumor markers

3 mL venous blood was collected from every fasting subject in the morning, and then the blood samples were stored at 37°C for 30 minutes, centrifuged for 10 minutes at 1500g. Finally, the serum was separated and stored at -80°C for follow-up analyses. Serum ProGRP and VEGF were determined by ELISA kits (Shanghai enzyme-linked biological technology company) according to the manufacturer’s instructions. Chemiluminescence detection kits (Beijing huaketai biotechnology company) were employed to detect serum CEA, CYFRA21-1, and NSE according to experimental procedures.

### Extraction of 22 lung CT nodule-based radiomic features

Radiomic features of lung CT nodules were extracted by MATLAB tool [27]. Firstly, the lung nodules on CT images were marked by three experienced radiologists. Then, threshold segmentation of pulmonary CT nodules was applied for the extraction of region of interest (ROI). Finally, 22 radiomic features of lung CT nodules were extracted. Among them, gray features included gray mean (f1), gray variance (f2), and gray histogram entropy (f3). Morphological features were consisted of seven order invariant distance (f4, f5, f6, f7, f8, f9, f10), calcification area (f11), calcification area/nodule area (f12), lobulation grade (f13), spiculation grade (f14), cavity number (f15), cavity area (f16) and cavity area/nodules area (f17). Texture features were composed of contrast (f18), correlation (f19), energy (f20), homogeneity (f21) and entropy (f22).

### Flow chart of proposed work

**Figure 1:**
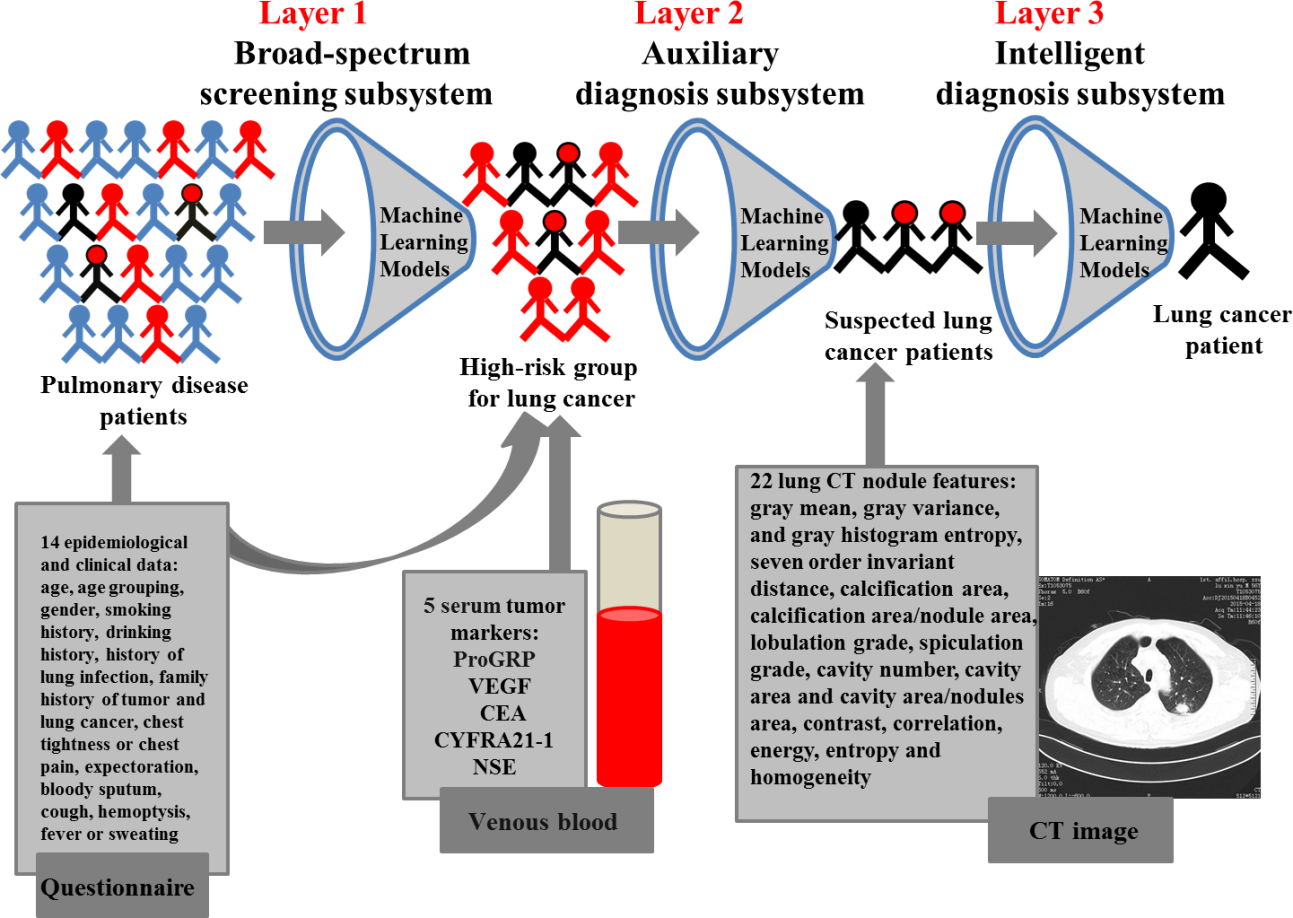
**A three-layer diagnosis system for lung cancer.**

A machine learning-based three-layer diagnostic system for lung cancer was proposed in this study as shown in Figure 1. The first layer was a broad-spectrum screening subsystem, which screened out the high-risk group of lung cancer from pulmonary disease patients. And, the machine learning-based screening models were developed using the 14 features of epidemiological characteristics and clinical symptoms. The high-risk individuals screened by the first-layer subsystem were included in the second-layer subsystem. The second layer was a machine learning-based auxiliary diagnosis subsystem constructed with the 14 features of epidemiological characteristics and clinical symptoms, and the 5 serum tumor markers for identifying suspected lung cancer patients from the high-risk groups. The suspected patients of lung cancer evaluated by the second-layer subsystem were further introduced into the third-layer subsystem. The third layer was an intelligent diagnosis subsystem, which was developed based on the 22 lung CT nodule-based radiomic features using machine learning models for further confirming lung cancer patients.

### Establishment of machine learning models

Based on the random sampling function of machine learning models, the samples were randomly divided into training set and testing set according to the ratio of 3:1 using partition node. The training set was employed to develop the models and testing set was used for evaluating the performance of the models. In each of the three subsystems, the 14 epidemiological characteristics and clinical symptoms were applied as the input variables for C5.0-1, ANN-1, and SVM-1 in the first-layer subsystem; The 14 epidemiological characteristics and clinical symptoms were combined with 5 serum tumor markers as the input variables for C5.0-2, ANN-2, and SVM-2 in the second-layer subsystem; The 22 lung CT nodule-based radiomic features were presented as the input variables for C5.0-3, ANN-3, and SVM-3 in the third-layer subsystem; While the groups (0 for lung benign diseases, 1 for lung cancer) were set as the output variables. Parameters for the models were set as follows:

### Configuration parameters of the C5.0 model

Use partitioned data: yes; Output type: Decision tree; Use boosting: yes; Number of trials: 9/25; Mode: Expert; Pruning severity: 75/25; Minimum records per child branch: 2; Use global pruning: yes; Use misclassification costs: yes; Model Evaluation: Calculate variable importance.

### Configuration parameters of the ANN model

Use partitioned data: yes; Method: Prune; Sample %:75.0; Accuracy:90.0%; Optimize: Memory; Use binary set encoding: yes; Show feedback graph: yes; Model selection: use best network; Mode: Expert; Hidden layers: Two or three (Layer 1: The number of variables. Layer 2: The number of features/2. Layer 3: 2). Model Evaluation: Calculate variable importance.

The input data of ANN were required to range from 0 to 1, so the parameters that did not meet this requirement were normalized using linear function to range from 0 to 1. Below was the formula:

Y=(X−Xmin)/(Xmax−Xmin)

(X was the original value, Y was transformed by the above formula via X, Xmax and Xmin were the maximum and minimum among all original data, respectively).

### Configuration parameters of the SVM model

Use partitioned data: yes; Mode: Sample/Expert; Stopping criteria: 1.0E-3; Regularization parameter (C): 9/1; Regression precision (epsilon): 0.1; Kernel type: Sigmoid/Polynomial; Bias: 0; gamma: 0.5; Model Evaluation: Calculate variable importance.

### Statistical analysis

Statistical analyses were performed by SPSS 21.0 software. SPSS Clementine 21.0 software was used for classification analysis. The data were expressed by Median (*P25*-*P75*) and analyzed with the Mann-Whitney U. Chi-Square test was employed for each contingency table. *P*-value of 0.05 was considered as a statistical test level.

Six indexes including accuracy, sensitivity, specificity, positive predictive value (PPV), negative predictive value (NPV), and area under the receiver operating characteristic curve (AUC) were used to evaluate the classification models.

## Supplementary Material

Supplementary Tables
