# Supplementary material for: Development of a machine learning-based multimode diagnosis system for lung cancer
Source: Aging (Albany NY). 2020 May 23;12(10):9840–54. doi: 10.18632/aging.103249 (PMC7288961; doi:10.18632/aging.103249)
Supplement: Supplementary Tables [file aging-12-103249-s001..pdf]

SUPPLEMENTARY TABLES

Supplementary Table 1. Demographic characteristics of subjects in the third-layer subsystem.

Variables	Lung benign (n=64)	Lung cancer (n=59)	χ^2/Z	*P*
Age Grouping				
≤45	12	1	9.447	0.002*
>45	52	58		
Age (year)	57(47-68)	59(53-68)	-1.371	0.170
Gender				
Female	33	42	4.968	0.026*
Male	31	17		
Smoking Status				
No	48	33	4.964	0.026*
Yes	16	26		
Drinking Status				
No	56	51	0.030	0.861
Yes	8	8		
History of Lung Infection				
No	44	34	1.637	0.201
Yes	20	25		
Chest Tightness or Chest Pain				
No	24	22	0.001	0.981
Yes	40	37		
Expectoration				
No	31	14	8.078	0.004*
Yes	33	45		
Bloody Sputum				
No	55	38	7.717	0.005*
Yes	9	21		
Cough				
No	21	8	6.316	0.012*
Yes	43	51		
Hemoptysis				
No	57	51	0.197	0.657
Yes	7	8		
Fever or Sweating				
No	35	43	4.380	0.036*
Yes	29	16		
Family History of Tumor				
No	62	50	5.546	0.019*
Yes	2	9		
Family History of Lung Cancer				
No	64	50	10.533	0.001*
Yes	0	9		

*: Statistically significant at P=0.05 level.

Supplementary Table 2. Comparison of AUCs among ANN, SVM and C5.0 models.

Comparison between models	*Z*	*P*
C5.0-1 vs ANN-1	1.981	0.048*
C5.0-1 vs SVM-1	2.114	0.035*
ANN-1 vs SVM-1	3.283	0.001*
C5.0-2 vs ANN-2	2.021	0.043*
C5.0-2 vs SVM-2	0.915	0.360
ANN-2 vs SVM-2	1.669	0.095
C5.0-3 vs ANN-3	0.035	0.972
C5.0-3 vs SVM-3	1.131	0.258
ANN-3 vs SVM-3	1.096	0.273

*: Statistically significant at P=0.05 level.
